# HAPPY TO CHAT – PROMOTING TALKING TO STRANGERS IN PUBLIC PLACES IN THE UK

**DOI:** 10.1093/geroni/igad104.3378

**Published:** 2023-12-21

**Authors:** Christina Victor, Dorothy Yen

**Affiliations:** Brunel University London, Uxbridge, England, United Kingdom; Brunel University London, Uxbridge, England, United Kingdom

## Abstract

After the Covid-19 pandemic, many countries are trying to reboot social connections in communities and societies, especially for older people who suffered mental wellbeing due to lockdowns and social distancing. Existing studies show talking to strangers can improve individuals’ well-being and reduce social isolations, although only with data from younger participants. This research aims to understand older people’s experience of talking to strangers, specifically in discussing how, why and where they feel most comfortable in talking to strangers. Using semi-structured interviews with 23 people (from 51 to 88 years old) based on their participation in a community-based Happy to Chat intervention (Happytochat.uk) from January to April 2023, we discuss how talking to strangers are consumed and practised by older adults. Applying agency and self-efficacy theory, findings show that their experience vary by age, gender and two types of self-efficacy: a) confidence in enjoying conversations with strangers; b) capability in navigating the risk involved in talking to strangers. The findings show that compared to the younger group (51-64 years old), the older participants (65+) are more willing and spend more time talking to strangers. Also, male participants tend to be more confident than females in navigating the risk associated with talking to strangers. Our research findings have practical and policy implications. Insights derived from the research findings are useful in developing training materials or conversation aids to improve current chatty schemes and increase older people’s self-efficacy in having conversations with strangers, thus benefiting from better social connections.

